# Lumican inhibits immune escape and carcinogenic pathways in colorectal adenocarcinoma

**DOI:** 10.18632/aging.202401

**Published:** 2021-01-20

**Authors:** Yiqing Zang, Qiuping Dong, Yi Lu, Kaiti Dong, Rong Wang, Zheng Liang

**Affiliations:** 1 Department of Otorhinolaryngology, Tianjin Medical University General Hospital, Tianjin 300052, China; 2 Department of Cancer Cell Biology, Tianjin’s Key Laboratory of Cancer Prevention and Therapy, National Clinical Research Center for Cancer, Tianjin Medical University Cancer Institute and Hospital, Tianjin 300060, China; 3 Department of Laboratory Medicine, Tianjin Medical University, Tianjin 300060, China

**Keywords:** lumican, colorectal adenocarcinoma, immune infiltration, miR200 family

## Abstract

Lumican (*LUM*), a small leucine-rich proteoglycan, is a component of the extracellular matrix. Abnormal *LUM* expression is potentially associated with cancer progression. In the present study, we confirmed high *LUM* mRNA expression in colorectal adenocarcinoma (COAD) through the UALCAN database. The Kaplan-Meier method, univariate, and multivariate COX analysis showed that high *LUM* expression is an independent determinant of poor prognosis in COAD. A COX regression model was constructed based on clinical information and *LUM* expression. The receiver operating characteristic (ROC) curve indicated that this model was highly accurate in monitoring COAD prognosis. The co-expression network of *LUM* was determined by LinkedOmics, which showed that *LUM* expression was closely related to immune escape and the miR200 family. Furthermore, we studied the co-expression network of *LUM* and found that *LUM* could promote tumor metastasis and invasion. The Tumor Immune Estimation Resource website showed that *LUM* was closely related to immune infiltration and correlated with regulatory T cells, tumour-associated macrophages, and dendritic cells. We found that *LUM* cultivated cancer progression by targeting the miR200 family to promote epithelial-to-mesenchymal transition. These findings suggest that *LUM* is a potential target for inhibiting immune escape and carcinogenic pathways.

## INTRODUCTION

Lumican (*LUM*) is a member of the small leucine-rich proteoglycan (SLRP) family, which is a component of the extracellular matrix. [1]. Its central region contains 10 leucine-rich repeats, which is one of the characteristics of the keratan sulfate subfamily [2]. *LUM* is associated with corneal disease and high myopia [3–11], cardiovascular disease [12–14], bone disease [15–18], polycystic ovary syndrome [19], and systemic lupus erythematosus [20]. There have been recent reports associating *LUM* with cancer. *LUM* may behave as an oncogene or tumor suppressor gene in several types of cancer, depending on the cellular context. For example, several studies demonstrated that high *LUM* expression is related to the unfavorable prognosis of breast cancer and pancreatic carcinoma [21, 22]. Further studies investigated the role of *LUM* in promoting epithelial-to-mesenchymal transition (EMT) in breast cancer [23, 24], and the depletion of *LUM* inhibited the proliferation and migration of bladder cancer cells by inactivating MAPK signaling [25]. High *LUM* expression was observed in drug-resistant ovarian cancer cell lines, suggesting its role in drug resistance [26]. Several experiments have shown that an increase of lumican expression in melanoma will reduce its growth and invasion [27]. Another study on melanoma showed that lumican was expressed in metastatic melanoma cells rather than normal melanocytes [28]. More recently, several studies confirmed that lumican was synthesized by dermal fibroblasts in malignant melanoma, and the decreased expression of lumican at the tumor margin may promote the proliferation of melanoma cells [29].

Colorectal cancer (CRC) is the third leading cause of global cancer mortality and is a serious threat to human health. According to the American Joint Committee on Cancer (AJCC), the five-year survival rate is almost 65% in Australia, Canada, the USA, and several European countries, but has remained < 50% in Asian and African countries [30–32]. Colorectal adenocarcinoma (COAD) accounts for more than 90% of CRC and is the predominant pathological type [33]. Over the past several years, only a few studies have focused on *LUM* in colon cancer. Immunohistochemistry studies showed that *LUM* was strongly expressed in colon cancer tumor cells, adjacent fibroblasts, and epithelial cells [34]. *LUM* expression had an unfavorable prognostic effect in patients with nodal metastasis [35]. *LUM* also increased during colorectal adenoma-to-carcinoma progression [36]. Functional experiments *in vitro* confirmed that *LUM* could enhance the migration of colon cancer cells [37]. However, the role of *LUM* in the COAD tumor microenvironment remains unclear.

In this study, we found that *LUM* expression was an independent negative prognostic factor of COAD by COX regression analysis. Furthermore, we studied the co-expression network of *LUM* and found that *LUM* could promote tumor metastasis and invasion. Through analysis of the immune microenvironment in the Tumor Immune Estimation Resource (TIMER) website, we found that *LUM* may prompt tumor immune escape. These results suggest that *LUM* is a potential novel target for COAD prognosis and treatment.

## RESULTS

### Elevated *LUM* expression in COAD

**Figure 1:**
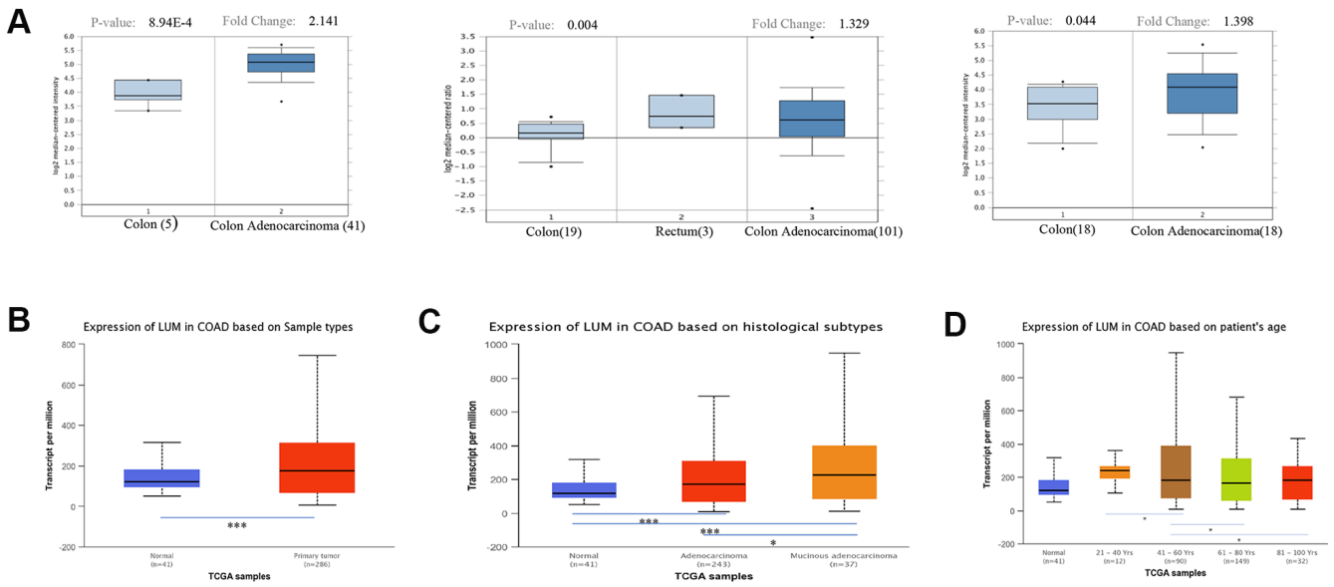
**Expression of *LUM* in cancer and paracancerous tissues of COAD in the Oncomine and UALCAN databases.** (**A**) mRNA expression of *LUM* in cancer tissues and paracancerous tissues in different microchips from the Oncomine database. Expression of *LUM* in (**B**) cancerous and paracancerous tissues, (**C**) cancer subtypes, and (**D**) by patient age. *P < 0.05, **P < 0.01, ***P < 0.001.

We initially evaluated *LUM* transcription in different databases to gain a relatively reliable result. According to the results of the Oncomine database, the mRNA expression of *LUM* was significantly higher in COAD tissues than in adjacent normal tissues for several studies (Figure 1A). The UALCAN database showed a similar result (Figure 1B). We also investigated whether *LUM* expression was correlated with different clinico-pathological characteristics. We found that *LUM* expression was higher in mucinous adenocarcinoma than in adenocarcinoma (P value < 0.05, Figure 1C). Our analysis of the UALCAN database indicated that *LUM* was downregulated for the 61-80 years age group, and upregulated in the 21-40 years and 81-100 years age groups compared to the 41-60 years age group (P < 0.05, Figure 1D). There was no statistically significant correlation between other clinical parameters (race, sex, weight, and lymph node metastasis status) and *LUM* expression in COAD patients (Supplementary Figure 1). As for the cancer stages, although *LUM* expression was higher in stage 3 compared to the stage 1 of COAD in the UALCAN database (P < 0.05), there was no significant difference in *LUM* expression between the different pathological stages of COAD in the GEPIA website (Supplementary Figure 2).

### The prognosis model of COAD

**Figure 2:**
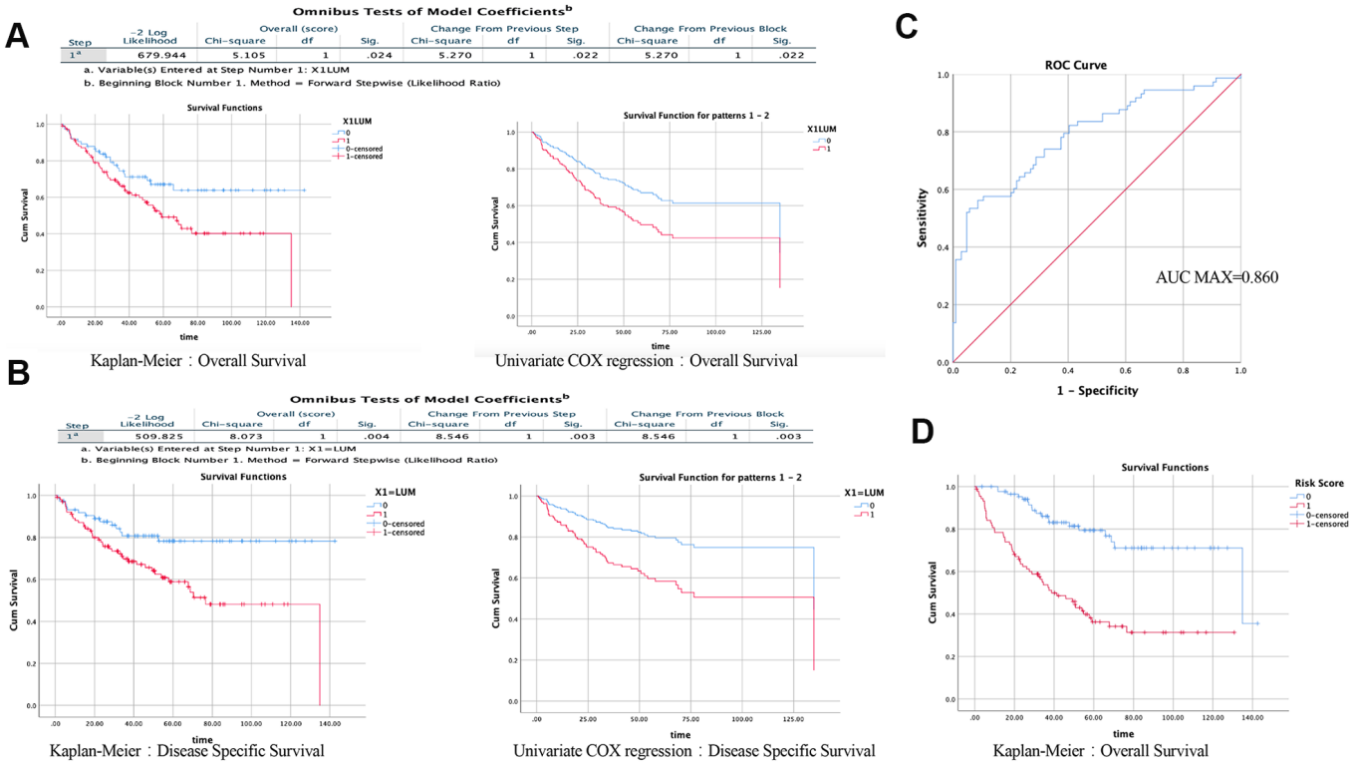
**Results of univariate and multivariate COX regression analysis in SPSS (GSE17536).** Effect of *LUM* on the prognosis of OS (**A**) and DSS (**B**) using the KM method and Univariate COX regression. (**C**) ROC analysis of the sensitivity and specificity of the risk score in predicting overall survival. (**D**) Effect of risk score on the prognosis of OS using the KM method.

We then used the Prognoscan website to obtain cohorts of COAD patients from the Gene Expression Omnibus (GEO) database (Supplementary Table 1). We excluded the cohorts with a small sample size or incomplete clinical information, and screened out a cohort (GSE17536). Baseline characteristics (age, sex, stage, grade, overall and disease-specific survival times) of 177 COAD patients are shown in Table 1. There were five cases with missing values. We performed a survival analysis using the Kaplan-Meier method and univariate COX regression based on *LUM* expression. Generally, high *LUM* expression was associated with poor overall survival (OS) (log-rank test, P = 0.024; univariate COX regression, P = 0.022) and disease-specific survival (DSS, log-rank test, P = 0.004; univariate COX regression, P = 0.003, Figure 2A, 2B). Besides *LUM*, other clinicopathologic characteristics such as age, gender, stage, and grade were independent but complementary prognostic factors. Moreover, multivariate analysis using the Cox proportional hazards model was performed to confirm the prognostic value of *LUM* mRNA expression. Prognostic factors after the univariate analysis were forwarded into the subsequent multivariate analysis and the five factors: *LUM*, age, gender, stage, and grade were included (Supplementary Table 2). Finally, three variables were included in the multivariate COX analysis. Only the *LUM* (HR = 1.887, 95% confidence interval (CI) [1.062–3.351], P = 0.030), age (HR = 1.025, 95% CI [1.005–1.046], P = 0.015), and stage (HR = 3.183, 95% CI [2.292–4.421], P-value < 0.001) were significantly associated with prognosis in multivariate analysis (Tables 2–3). These results indicate that *LUM* mRNA expression is an independent prognostic factor and increased *LUM* mRNA levels were associated with poor OS.

**Table 1:** Clinical parameter of GSE17536.

**Stage**	**I**	**II**	**III**	**IV**
**N**	24	57	57	39
**Grade**	1	2	3	
**N**	16	134	27	
**Gender**	0	1		
**N**	81	96		
**Age**	<65	>=65		
**N**	78	99		
**OS-Status**	0	1		
**N**	104	73		
**DSS-Status**	0	1		
**N**	122	55		

N=number; Age is divided into two groups according to the average; Status (0) = survival, status (1) = dead.

**Table 2:** Results of multivariate regression analysis: results of variable analysis included in the model.

**Variables in the equation**									
								**95.0%CI for exp(B)**	
		**B**	**SE**	**Wald**	**df**	**Sig.**	**Exp(B)**	**Lower**	**Upper**
Step 1	X4=Stage	1.049	0.154	46.482	1	0.000	2.854	2.111	3.859
Step 2	X2=Age	0.020	0.010	4.113	1	0.043	1.021	1.001	1.041
	X4=Stage	1.097	0.155	49.917	1	0.000	2.996	2.210	4.062
Step3	X1=LUM	0.635	0.293	4.691	1	0.030	1.887	1.062	3.351
	X2=Age	0.025	0.010	5.889	1	0.015	1.025	1.005	1.046
	X4=Stage	1.158	0.168	47.692	1	0.000	3.183	2.292	4.421

**Table 3:** Results of multivariate regression analysis: results of omnibus tests about model coefficients.

**Omnibus tests of model coefficients^d^**										
		**Overall (score)**			**Change from previous step**			**Change from previous block**		
**Step**	**-2Log Likelihood**	**Chi-square**	**df**	**Sig.**	**Chi-square**	**df**	**Sig.**	**Chi-square**	**df**	**Sig.**
1^a^	630.754	51.546	1	0.000	54.459	1	0.000	54.459	1	0.000
2^b^	626.462	56.197	2	0.000	4.292	1	0.038	58.751	2	0.000
3^c^	621.475	57.306	3	0.000	4.988	1	0.026	63.739	3	0.000

a. Variable(s) Entered at Step Number 1:X4=Stage.

b. Variable(s) Entered at Step Number 2:X2=Age.

c. Variable(s) Entered at Step Number 3:X1=LUM.

d. Beginning Block Number 1. Method =Forward Stepwise(Likedlihood Ratio).

Therefore, to accurately evaluate the prognosis of COAD patients, a prognostic prediction model is needed. According to the results of the multivariate Cox regression analysis, the formula is: Risk Score = [mRNA level of LUM*0.635] + [age*0.02)] + [stage*1.158]. To assess the reliability of the formula established herein, the receiver operating characteristic (ROC) curve was generated, and the AUC was calculated (AUC = 0.790; AUC max = 0.860; P < 0.001, Figure 2C). The area under the ROC curve (i.e., overall ability of *LUM* to discriminate between controls and patients) was 0.790 (95% CI [0.721–0.860]; z test P < 0.001). The results showed that this formula could predict the prognosis of patients with COAD. To determine the performance of the Risk Score in predicting clinical outcomes, the Kaplan-Meier survival curve was plotted to analyze different survival times between high- and low-risk groups. The results showed that the prognosis of patients in the high-risk group was worse than that in the low-risk group (log rank P value < 0.001, Figure 2D). These findings indicate that the Risk Score based on *LUM* has potential for predicting COAD survival.

### *LUM* co-expression networks in COAD

**Figure 3:**
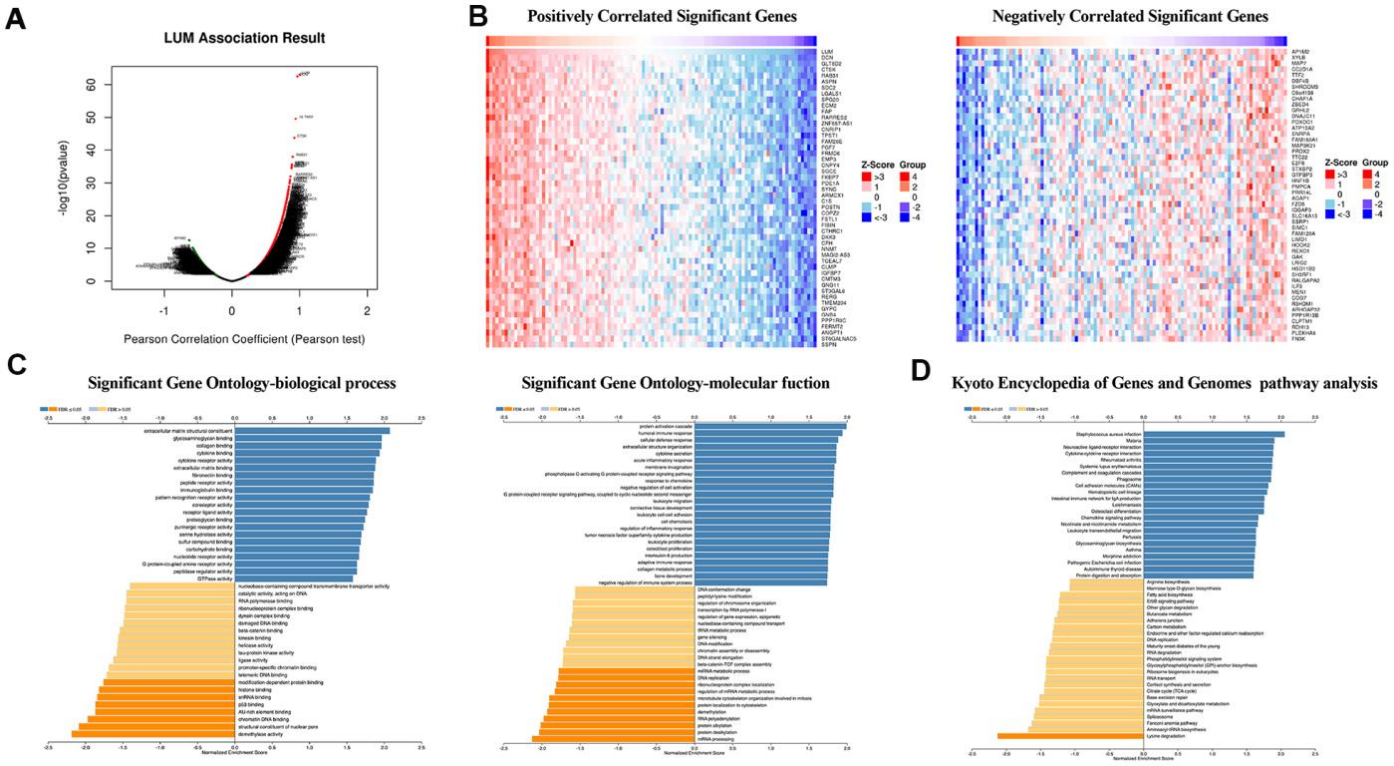
**LUM co-expression networks in COAD (LinkedOmics).** (**A**) Volcanic diagrams of positively and negatively correlated genes. (**B**) Heat map of positively and negatively correlated TOP50 genes. (**C**) GO and (**D**) KEGG pathway analysis of related genes.

**Figure 4:**
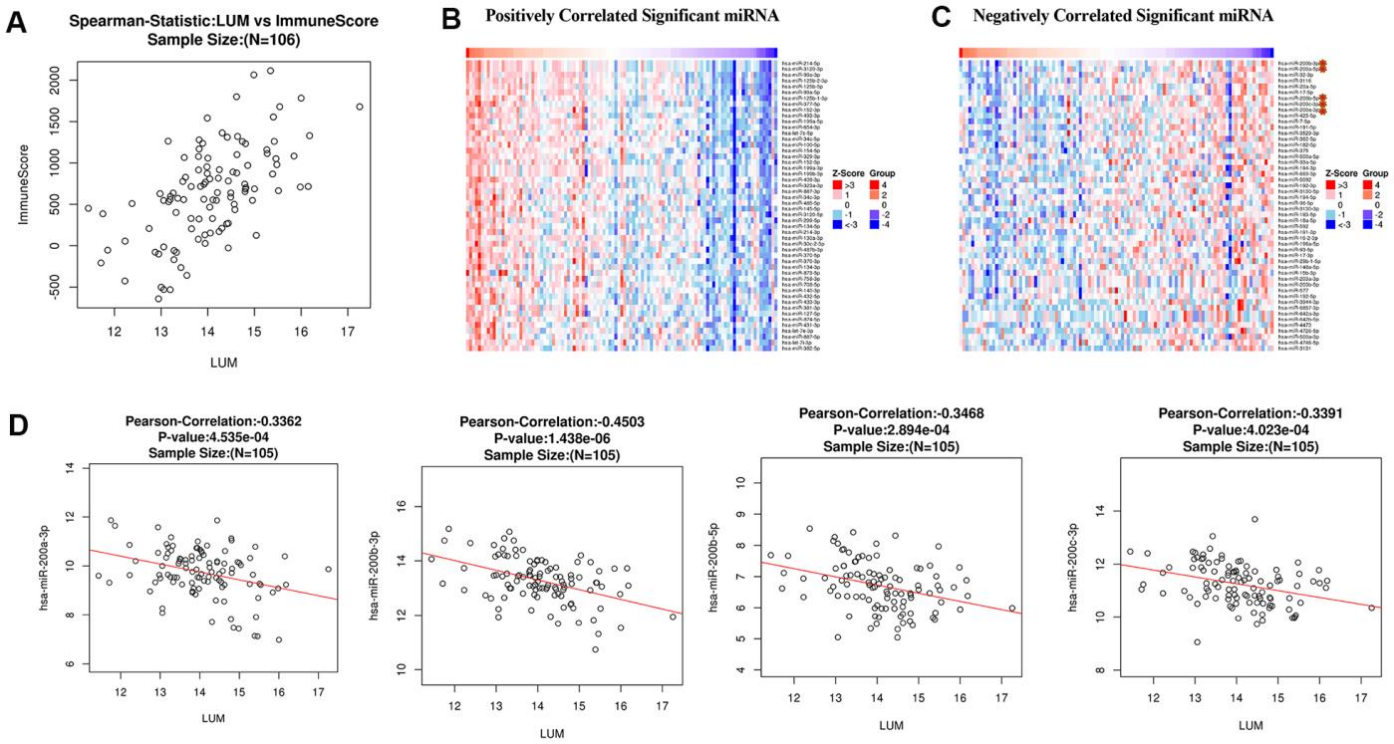
**Analysis of *LUM* in COAD.** (**A**) Analysis of the correlation between *LUM* and immunity (ImmuneScore = 0.6042, P < 0.001, and FDR = 1e-08). Heat map of miRNA (**B**) positively and (**C**) negatively related to *LUM* (TOP50). (**D**) Scatter diagram of the relationship between *LUM* and the miR200 Family.

To further explore the *LUM*-related molecules functioning in COAD, we used the LinkedOmics database to analyze mRNA /miRNA sequencing and clinical data from 105 COAD patients in the clinical proteomic tumor analysis consortium (CPTAC) database. As shown in the volcano plot, there are 2,427 and 2,011 significant positive and negative correlation genes (red and green dots), respectively, with *LUM* by the LinkFinder module (false discovery rate (FDR) < 0.01, Figure 3A). The total genes co-expressed with *LUM* are shown in Supplementary Table 3. The first 50 significant genes with positive and negative correlation with *LUM* in COAD (FDR < 0.05) are shown in the heat map in Figure 3B. Significantly enriched GO annotations were analyzed by Gene set enrichment analysis (GSEA) in the linkInterpreter module. The genes associated with *LUM* were mainly located in biological processes that are involved with protein activation cascades, humoral immune response, cellular defense response, protein alkylation, RNA polyadenylation, and protein dealkylation (Figure 3C). The molecular function was involved in extracellular matrix (ECM) structural constituents, immunoglobulin binding, nucleotide receptor activity, demethylase activity, structural constituents of nuclear pores, and p53 binding (Figure 3C). KEGG pathway analysis revealed enrichment in immune response, cell adhesion pathways, and lysine degradation (Figure 3D). Thus, we analyzed the correlation between *LUM* and immune score, and found that there was a significant correlation between *LUM* and immunity (P < 0.001, Figure 4A).

MicroRNAs regulate gene expression at the post-transcriptional level by binding to mRNA and inducing translational repression. Many dysregulated miRNA in human cancer have carcinogenic or tumor suppressive activity [38–40]. To further build the co-expression networks, we analyzed the positively and negatively correlated miRNAs (TOP50) of *LUM* in COAD by LinkedOmics (Figure 4B, 4C). All relevant miRNAs are listed in Supplementary Table 4. Then, we analyzed the TOP10 miRNAs that were positively or negatively correlated with *LUM*. The miR125b family (including miR125b-2-3p, miR125b-5p, miR125b-1-3p) was positively correlated with *LUM* expression (Supplementary Figure 3). There are papers regarding miR125b in human colon cancer, but they play different roles for different situations [41, 42]. Therefore, we did no further analysis of it. The miR200 family (including miR200a-3p, miR200b-3p, miR200b-5p, miR200c-3p) was the most significantly negatively correlated miRNA family (Figure 4D). The miR200 family has a central role in EMT and potential for both prognostic and therapeutic management of CRC [43]. Therefore, we needed to further study the relationship between the miR200 family and *LUM* to explore the role of *LUM* in EMT.

### *LUM* expression is correlated with immune infiltration level in COAD

**Figure 5:**
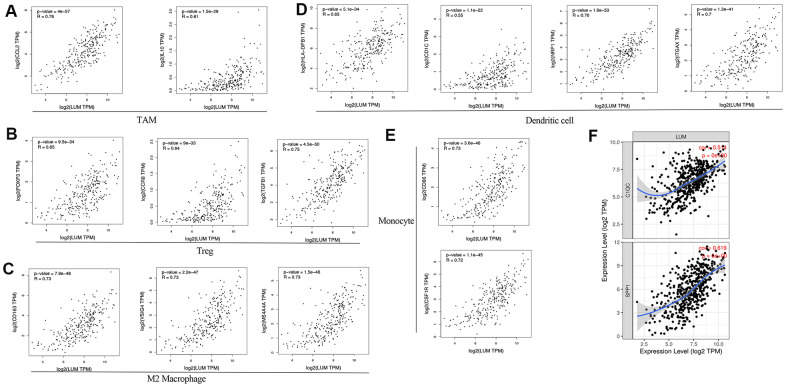
***LUM* expression correlated with macrophage polarization in COAD.** Markers include (**A**) *CCL2* and *IL10* of TAMs (tumor-associated macrophages); (**B**) *FOXP3*, *CCR8*, and *TGFB1* of Tregs; (**C**) *CD163*, *VSIG4*, and *MS4A4A* of M2 macrophages; (**D**) *HLA-DPB1*, *CD1C*, *NRP1*, and *ITGAX* of DCs; (**E**) *CD86* and *CSF1R* of monocytes; (**F**) *C1QC* and *SPP1* of TAM subtypes.

Tumor-infiltrating lymphocytes are an independent predictor of sentinel lymph node status and cancer survival [44–46]. Therefore, we investigated whether *LUM* expression was correlated with immune infiltration levels in COAD. First, we evaluated the correlation between *LUM* expression and the level of COAD immune infiltration from the TIMER website. The analysis showed that *LUM* expression in COAD was positively correlated with the infiltration level of B cells (r = 0.122, P=0.0140), CD8+ T cells (r = 0.308, P < 0.001), CD4+ T cells (r = 0.314, P < 0.001), macrophages (r = 0.577, P < 0.001), neutrophils (r = 0.482, P < 0.001), and dendritic cells (DCs, r = 0.475, P < 0.001, Supplementary Figure 4). After purity correlation adjustments, the results revealed that *LUM* expression was significantly correlated with most immune marker sets of various immune and T cells. Moreover, we found that most of the marker sets of monocytes, tumor-associated macrophages (TAM), M2 macrophages, and DC markers were strongly correlated with the *LUM* expression in COAD. Table 4 shows that the expression of *CCL-2* and *IL10* in TAMs [47], *CD86* and *CD115* in monocytes, *HLA-DPB1*, *BDCA-1*, *BDCA-4*, and *CD11c* in DCs [41]; *CD163*, *VSIG4,* and *MS4A4A* in M2 macrophages [42–44]; and *CCR8*, *TGFB1*, and *STAT5B* in regulatory T cells (Treg) [48, 49] are significantly correlated with *LUM* in COAD (P < 0.001). We further analyzed the correlation between *LUM* expression and the above markers in the GEPIA database. We found that there was a significant correlation between *LUM* and the expression of these immune infiltrating cell markers in COAD (Figure 5), such as *HLA-DPB1,* *BDCA-1*, *BDCA-4*, and *CD11c.* These results suggest that *LUM* regulates macrophage polarization in COAD. In addition to Treg cells, *LUM* was positively correlated with *FOXP3*, *CCR8*, and *TGFB1* in COAD. *FOXP3* plays an critical role in Treg cells, allowing acquisition of full suppressive function and stability for the Treg lineage, thus inhibiting cytotoxic T cells attacks on tumor cells [50]. DCs can promote tumor metastasis by increasing the cytotoxicity of Treg cells while reducing the cytotoxicity of CD8+ T cells [32]. There was also a significant correlation between *LUM* and T cell depletion genes, such as *PD-1*, *CTLA4*, *LAG3*, and *TIM-3* in COAD (Table 4). These results indicate that the high *LUM* expression plays a crucial role in T cell depletion. Recently, TAMs have been subdivided into subsets called *C1QC +* and *SPP1+* by single cell sequencing [51]. We found that *LUM* is more related to *SPP1+* than *C1QC +* TAMs (Figure 5F), implying that *LUM* may exert more effects on the *SPP1+* subset. In summary, these results imply that *LUM* plays a crucial role in immune escape within the colon cancer microenvironment.

**Table 4:** Correlation analysis between LUM and relate genes and markers of immune cells in TIMER.

**Description**	**Gene markers**	**COAD**		
		**None**		**Purity**	
		**cor**	**P**	**cor**	**P**
B cell	CD19	0.213	***	0.107	3.12E-02
	CD79A	0.335	***	0.215	***
Tcell(general)	CD3D	0.261	***	0.142	*
	CD3E	0.334	***	0.22	***
	CD2	0.381	***	0.298	***
CD8+T cell	CD8A	0.291	***	0.18	**
	CD8B	0.167	**	0.11	2.60E-02
Monocyte	CD86	0.671	***	0.63	***
	CD115(CSF1R)	0.567	***	0.491	***
TAM	CCL2	0.736	***	0.684	***
	CD68	0.381	***	0.315	***
	IL10	0.523	***	0.488	***
M1 Macrophage	INOS (NOS2)	-0.212	***	-0.26	***
	IRF5	0.231	***	0.232	***
	COX2(PTGS2)	0.291	***	0.226	***
M2 Macrophage	CD163	0.636	***	0.577	***
	VSIG4	0.613	***	0.552	***
	MS4A4A	0.638	***	0.591	***
Neutrophils	CD66b (CEACAM8)	-0.156	**	-0.14	*
	CD11b (ITGAM)	0.629	***	0.571	***
	CCR7	0.331	***	0.22	***
Natural killer cell	KIR2DL4	0.1	3.33E-02	0.004	9.33E-01
	KIR2DL3	0.081	8.50E-02	0.03	5.43E-01
	KIR3DL3	-0.026	5.80E-01	-0.035	4.81E-01
	KIR3DL2	0.152	*	0.066	1.84E-01
	KIR2DS4	0.143	*	0.104	3.71E-02
	KIR2DL1	0.139	*	0.086	8.47E-02
	KIR3DL1	0.154	**	0.092	6.48E-02
Dendritic cell	HLA-DPB1	0.462	***	0.375	***
	HLA-DQB1	0.288	***	0.199	***
	HLA-DRA	0.479	***	0.395	***
	HLA-DPA1	0.486	***	0.403	***
	BDCA-1(CD1C)	0.448	***	0.368	***
	BDCA-4(NRP1)	0.732	***	0.698	***
	CD11c (ITGAX)	0.599	***	0.54	***
Th1	T-bet (TBX21)	0.303	***	0.220	***
	STAT4	0.383	***	0.299	***
	STAT1	0.388	***	0.339	***
	IFN-γ (IFNG)	0.203	***	0.162	1.01E-02
	TNF-α (TNF)	0.336	***	0.288	***
Th2	GATA3	0.375	***	0.313	***
	STAT6	-0.229	***	-0.246	***
	IL13	0.312	***	0.245	***
	STAT5A	0.124	*	0.09	0.0714
Tfh	BCL6	0.381	***	0.296	***
	IL21	0.229	***	0.196	***
Th17	STAT3	0.223	***	0.161	*
	IL17A	-0.117	1.25E-02	-0.127	1.02E-02
Treg	FOXP3	0.494	***	0.413	***
	CCR8	0.548	***	0.491	***
	TGFβ (TGFB1)	0.563	***	0.488	***
	STAT5B	0.171	**	0.182	*
T cell exhaustion	PD-1 (PDCD1)	0.261	***	0.153	*
	CTLA4	0.382	***	0.301	***
	LAG3	0.243	***	0.141	*
	TIM-3 (HAVCR2)	0.667	***	0.631	***
	GZMB	0.070	1.33E-01	0.046	3.55E-01

P>0.05:gray color; P<0.05:*; P<0.01:**;P<0.001:***.

### *LUM* targets the miR200 family and its downstream signaling pathways

**Figure 6:**
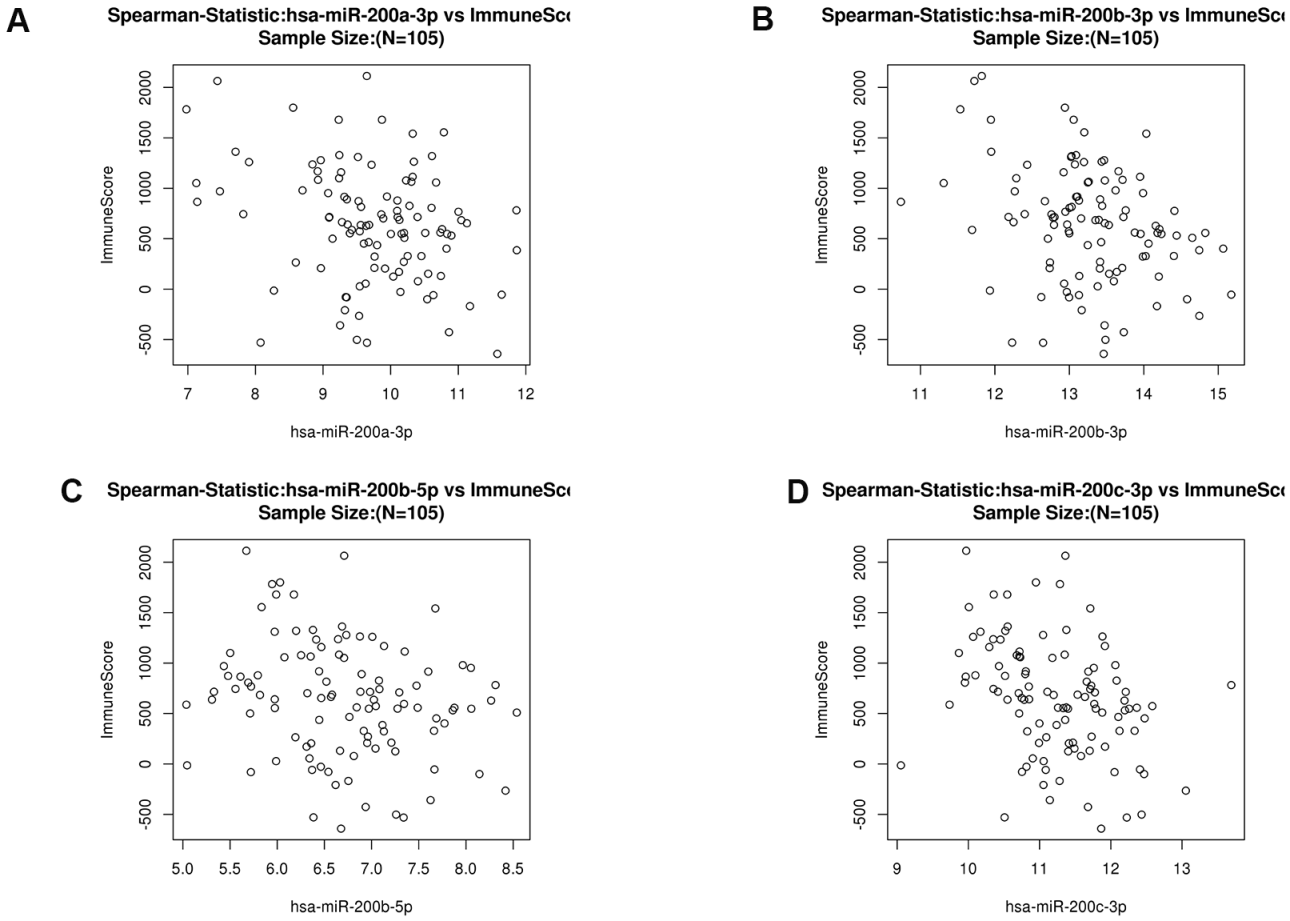
Scatter diagram of the relationship between immune score and the miR200 family (hsa-miR-200a-3p (**A**), hsa-miR200b-3p (**B**), hsa-miR200b-5p (**C**) and hsa-miR-200c-3p (**D**) in COAD.

**Figure 7:**
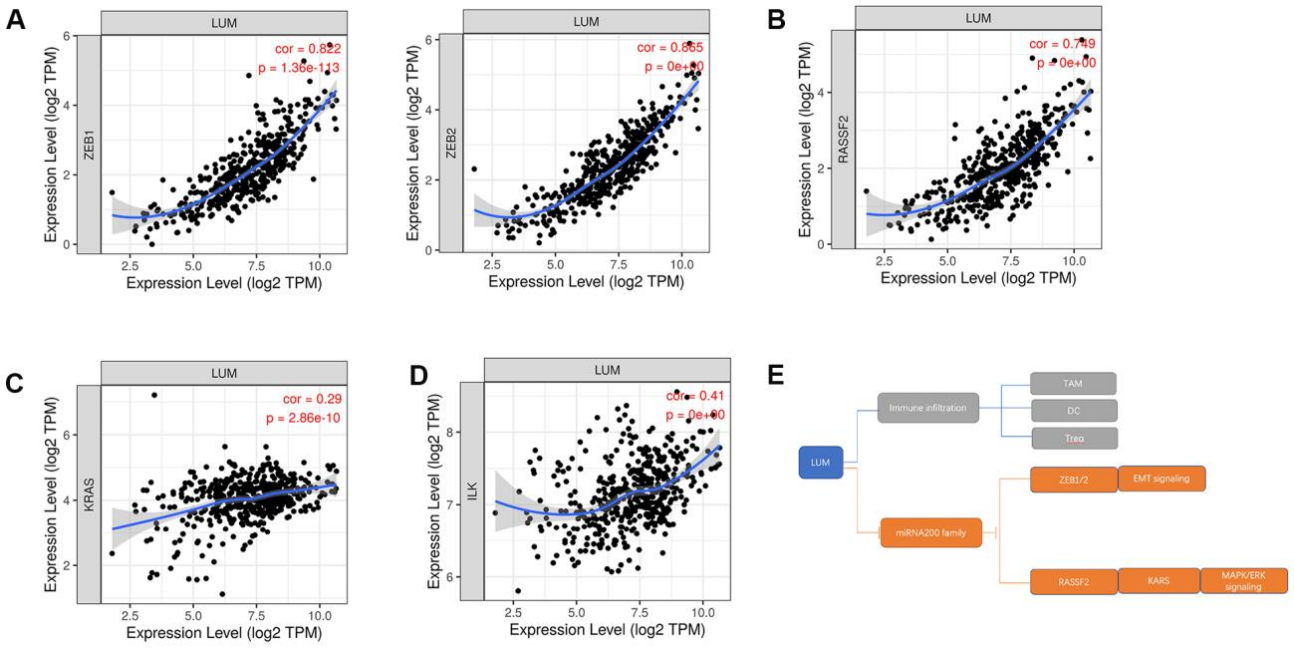
**The relationship between *LUM* and downstream genes of the miR200 family in COAD.** Scatter diagrams of the relationship between *LUM* and (**A**) *ZEB1*, *ZEB2*; (**B**) *PASSF2*; (**C**) *KRAS*; (**D**) *ILK*. (**E**) Pattern diagram of targeting the miR200 family and its downstream pathways for *LUM*.

To further explore the targets of *LUM* in COAD, we found a significant negative correlation between the expression of miR200 family and *LUM* through LinkedOmics. There was a significant positive correlation between *LUM* and immunity (Figure 4). The scatter plot results show that the miR200 family had no significant relation with immunity in COAD (Figure 6). Therefore, it is not possible for miR200 to regulate signaling pathways upstream of *LUM*. According to the negative correlation between them, miR200 can be targeted for *LUM*. Thus, we detected the relationship between *LUM* and miR200's downstream genes. Previous studies had confirmed that the miR200 family regulated EMT to enhance tumor migration and invasion [43, 52, 53]. It has been demonstrated that the miR200 family suppresses EMT through the transcriptional repressors *ZEB1* and *ZEB2* [54, 55]. The miR200 family also affects cell proliferation by regulating *RASSF2* expression, a negative regulator of *KRAS*, then subsequently enhancing the *KRAS/MAPK/ERK* signaling pathways [56–58]. Accordingly, we used the TIMER website to analyze the correlation between *LUM* and miR200's downstream genes, including *ZEB1*, *ZEB2*, *RASSF2,* and *KRAS*. *ILK* is a marker EMT signaling pathway activation in CRC [59]. We found that *LUM* had a strong positive correlation with *ZEB1* (cor = 0.822, P < 0.001), *ZEB2* (cor = 0.855, P = 0e+00), and *RASSF2* (cor = 0.749, P = 0e+00, Figure 7A, 7B). There was a statistically significant positive correlation between *KARS* (cor = 0.29, P < 0.001), *ILK* (cor = 0.41, P = 0e+00), and *LUM* (Figure 7C, 7D). This result is consistent with the negative regulation of these genes by the miR200 family. Therefore, we inferred that *LUM* promotes tumor invasion and migration by targeting the miR200 family and regulating its downstream signaling pathways (Figure 7E).

## DISCUSSION

Although a few studies have maintained that *LUM* is associated with unfavorable colon cancer progression, its potential functions and regulatory network in COAD has not been analyzed. According to the TNM classification system of the AJCC and the International Union for Cancer Control (UICC), the prognosis of patients with resectable CRC depends on the histopathological criteria of tumor invasion and the characteristics of tumor cell differentiation [60, 61]. However, this system is useful but incomplete for prognostic information [62]. Therefore, a novel prognostic and predictive marker is required for COAD. In this study, we combined public database resources with different analysis methods to investigate *LUM* on survival, co-expression networks, and tumor-associated immunity.

From the Oncomine and UALCAN databases, we found that the expression of *LUM* in COAD was significantly higher than adjacent tissues. The results of Kaplan-Meier and COX univariate analysis revealed that high *LUM* expression had a worse prognosis in COAD. Then, *LUM* expression, age, and stage were used in the COX multivariate regression model to establish a predictive model for calculating the risk scores of COAD patients. The patients in the analyzed sample were divided into high- and low-risk groups, which was based on the risk scores. To assess the reliability and efficiency of risk score in terms of survival prediction, ROC curve analysis and KM plot analysis were performed. The results showed that the risk score could predict the prognosis of COAD patients. Thus, our research demonstrates that *LUM* expression is an independent negative prognostic biomarker in COAD patients.

To understand the role of *LUM* in the colon cancer microenvironment, we analyzed the co-expression network of *LUM* in the LinkedOmics website. Our results suggest that *LUM* is associated with immune infiltration and the miR200 family, thus providing a new possible target for COAD treatment. The results indicated that the function of *LUM* co-expression genes were enriched for immunity and cell adhesion by GO analysis. Through the LinkFinder module, there was a positive correlation between *LUM* and immune score, indicating that the function of *LUM* was related to immune cell infiltration and metastasis. Then, we used the TIMER website and found that *LUM* expression was positively correlated with different immune cell infiltration and markers (monocytes, TAMs, macrophages, DCs, and Treg cells). This suggests that *LUM* has a crucial role in evading immunity and metastasis by reducing the cytotoxicity of CD8+ T cells and increasing T cell depletion in COAD. *LUM* is an extracellular proteoglycan and a class II SLRP. Proteoglycans are one of the major ECM components and play a critical role in tissue homeostasis and immunity. Changes in proteoglycan expression in tumor cells and the tumor microenvironment is related to oncogenesis [63]. Cancer immunoediting consists of three stages: elimination (cancer immune surveillance), balance, and escape. Tumor ECM contributes to the development of an immunosuppressive network. ECM remodeling involves cytokines and chemokines that allow tumor immune escape. Tumor-extracellular matrix interactions and matrix remodeling are necessary for promoting a tumor immune response. Therefore, proteoglycans are attractive pharmacological targets in cancer [64, 65]. SLRP was initially associated with regulating the innate immune response, and triggering these responses can initiate tumorigenesis [66]. Therefore, high *LUM* expression may play a promoting role in tumorigenesis and immune escape of COAD, which could result in poor patient outcomes.

We also analyzed co-expression in *LUM* miRNAs and found that the most significant TOP10 negative correlation miRNA was the miR200 family members. We excluded the possibility of the miR200 family as upstream of *LUM*, rather, *LUM* could be upstream of the miR200 family. Then we explored the relationship between *LUM* and downstream of the miR200 family. The results showed that *LUM* was highly correlated with *miRNA200/ZEB/EMT* signaling pathways, indicating that *LUM* promotes EMT by targeting miRNA200. We also found that there is a significant correlation between *LUM* and the *miRNA200/RASSF1/KARS/MAPK/ERK* signaling pathway. The EMT and *MAPK/ERK* pathways are related to carcinogenesis and development of colon cancer. The miR200 family regulates EMT through the *ZEB1/E-Cadherin* signaling pathway. *ZEB1* is a transcriptional repressor of the miR200 family. Therefore, it can be assumed that *LUM* activates *ZEB1* to transcriptively inhibit expression of the miR200 family and regulate its downstream pathways. Previous studies revealed abnormal silencing of the miR200 family in tumors is caused by abnormal DNA methylation [67]. High expression of *LUM* can inhibit DNA demethylation activity by GO enrichment analysis. We speculate that *LUM* maintains the abnormal DNA methylation state of miR200 family by inhibiting DNA demethylation, thus silencing expression of the miR200 family. Therefore, *LUM* is crucial for evading immunity and is also related to the carcinogenic pathway. This makes *LUM* a target with great therapeutic potential.

At present, there are a variety of strategies for treating advanced COAD, but the outlook remains poor for most patients. In recent years, emerging immunomodulatory antibodies targeting PD-1, PD-L1, and CTLA-4 have rapidly been developed. However, the efficacy of using them for COAD remains controversial [68, 69]. Depleted immunosuppressant TAMs is another emerging therapy to promote anti-tumor immune response. There are two main kinds of blockers for CSF1R and CD40. When the CSF1R blocker is used alone, it could lead to the recruitment and proliferation of FOXP3+ Treg cells and macrophages, which has an effect on C1QC+TAM, but little effect on SPP1+TAM [70–73]. C1QC+TAM is related to inflammation while SPP1+TAM is related to metastasis and angiogenesis of CRC [51]. In our study, the expression of LUM was positively correlated with FOXP3+ Treg and promoted the polarization of macrophages. Moreover, in terms of the influence on the subtypes of TAM, LUM is more related to SPP1+TAM than C1QC+TAM. Therefore, LUM blockers could be used together with CSF1R blockers to make up for the defects of CSF1R blockers and improve the immune therapy effect. It is also well established that colon cancer occurrence is closely related to EMT. EMT is a dominant program in human colon cancer [74]. Our study results reveal that LUM may target the miR200 family to regulate the EMT pathway. Several studies confirmed that a selective ECM inhibitor can control tumor metastasis [75]. Thus, a LUM inhibitor could participate in immunotherapy and inhibit EMT at the same time. Unfortunately, there were no targeted drugs found for LUM in three drug target databases (DrugBank, Potential Drug Target Database, and Therapeutic Target Database). Previous preclinical studies, vaccines, CAR-T-NK cells, monoclonal antibodies, immunotoxin-targeted proteoglycans and their ligands, enzymes, receptors, and signal molecules have shown encouraging results in the synthesis, accumulation, and degradation of proteoglycans [64]. Therefore, these techniques can be used for LUM inhibitors. In the JASPAR website, we predicted that SP3 was bound to the GC box in the promoter region (Supplementary Figure 5). Grover et al. analyzed the promoter sequence of LUM and found that SP3 binded to the GC box in the promoter region to transcriptively activate LUM [76]. Mitramycin A (MTM-A) is an anti-tumor antibiotic and frequently used in clinical chemotherapeutic drugs. MTM-A preferentially binds to the GC-rich sequence in DNA, competitively blocks the binding of Sp TFs to the GC box in the gene promoter, and inhibits the transcription of Sp-regulated genes [77–80]. We speculate that MTM-A may be used as a targeting drug for LUM in the future. It could be used in combination with CSF1R blockers as an immunotherapy, and, simultaneously, as an inhibitor of EMT signaling pathways in cancer the miR200 family.

In conclusion, this study provides evidence for the crucial role of *LUM* in the prognosis and carcinogenesis of COAD. Our results suggest that *LUM* may be a novel target that can inhibit both immune escape and carcinogenic pathways. However, further experiments *in vitro* and *in vivo* should focus on the molecular mechanisms underlying the involvement of *LUM* in COAD.

## MATERIALS AND METHODS

### Oncomine database analysis

Oncomine (https://www.oncomine.org/resource/login.html) is one of the largest oncogene chip databases and integrated data mining platforms in the world. It integrates GEO, TCGA, RNA, and DNA-SEQ data from published literature [81]. We used its online analysis tool to analyze the expression of *LUM* in COAD and paracancerous tissues in the tumor database.

### UALCAN database analysis

UALCAN (http://ualcan.path.uab.edu) used TCGA Level 3 RNA-Seq and clinical data from 31 cancer types [82]. We used it to analyze the relative expression of *LUM* in tumor and normal samples and different tumor subgroups based on cancer stage, tumor grade, race, weight, or other clinicopathological features of COAD. A t-test was used to determine the statistical significance between different levels of *LUM* expression.

### PrognoScan database analysis

PrognoScan (http://dna00.bio.kyutech.ac.jp/PrognoScan/index.html) has a large collection of publicly available cancer microarray data sets with clinical annotations [83]. We used it to find the gene chip of COAD. The sample quantity of GSE12945 and GSE17537 was less than 100, and GSE14333 lacked of overall survival information. Therefore, we excluded these datasets.

### Establishment of a multivariate COX regression model

The gene expression dataset of primary colorectal tumors (GSE17536) was downloaded from the GEO database. IBM SPSS Statistics for Mac 26.0 software was used to process the data. We first averaged *LUM* expression, then divided it into two groups (cut-off value = 12.0869): high and low *LUM* expression, and then made the survival curve with the survival times of OS and DSS (Kaplan-Meier method and Univariate COX regression method). Then, the patient's clinical parameters (age, grade, stage, gender) was added, and the prognosis model was obtained by COX multivariate regression analysis (P < 0.05). Through the formula, we obtained the risk score value and patient survival to analyze the ROC curve and verify the prediction accuracy of the formula (AUC > 0.70). Then, according to the mean risk score, we divided the patients into high and low risk groups for Kaplan-Meier survival analysis (P < 0.05).

### LinkedOmics website analysis

LinkedOmics database (http://www.linkedomics.org) contains multiple data sets and clinical data from 32 cancer types in 11,158 patients from the Cancer Genome Map (TCGA) project [84]. The LinkFinder module of LinkedOmics was used to study genes differentially expressed in correlation with LUM in the TCGA COAD cohort (n = 105). The results were analyzed statistically using Pearson’s correlation coefficient. We use the LinkFinder module to obtain volcanic maps, heat maps, and tables of genes and miRNA that are positively and negatively related to LUM (Pearson’s correlation). The scatter diagrams of gene and immune scores were also obtained (Non-parametric analysis; P < 0.001). We used the LinkInterpreter module to get the GSEA analysis of the co-expression network and the functional enrichment analysis of related genes and miRNAs. Minimum number of genes (Size = 3; Simulations = 1000; HDR < 0.05)

### TIMER website analysis

TIMER pre-calculated the levels of six tumor-infiltrating immune subsets from 10,897 tumors from 32 cancer types to comprehensively study the molecular characteristics of tumor-immune interactions [85]. We used the gene module to see the correlation between genes and the level of immune infiltration. We used the correlation module to examine the correlation of different genes in COAD (Spearman correlation analysis). The partial Spearman correlation of tumor purity correction calculated the correlation between *LUM* expression and immune genes, and controlled the tumor purity.

### GEPIA database analysis

GEPIA (http://gepia.cancer-pku.cn/index.html) is a website that provides fast and customizable functionality based on TCGA and GTEx data [86]. We used multiple gene analysis-correlation modules to examine the correlation of different genes in COAD and paracancerous tissues (Pearson correlation analysis).

### Statistical analysis

The survival curve was derived from the Kaplan–Meier method; the log-rank test was used to compare the survival rate. A Cox proportional hazards model was used to calculate the adjusted hazard ratio (HR) with a 95% confidential interval (95% CI). The ROC curve and the area under ROC curve (ROC-AUC) were also calculated to evaluate the predictive ability of built model (AUC > 0.70).

## Supplementary Material

Supplementary Figures

Supplementary Table 1

Supplementary Table 2

Supplementary Table 3

Supplementary Table 4
