# Supplementary material for: Lumican inhibits immune escape and carcinogenic pathways in colorectal adenocarcinoma
Source: Aging (Albany NY). 2021 Jan 20;13(3):4388–408. doi: 10.18632/aging.202401 (PMC7906189; doi:10.18632/aging.202401)
Supplement: Supplementary Figures [file aging-13-202401-s001.pdf]

SUPPLEMENTARY FIGURES

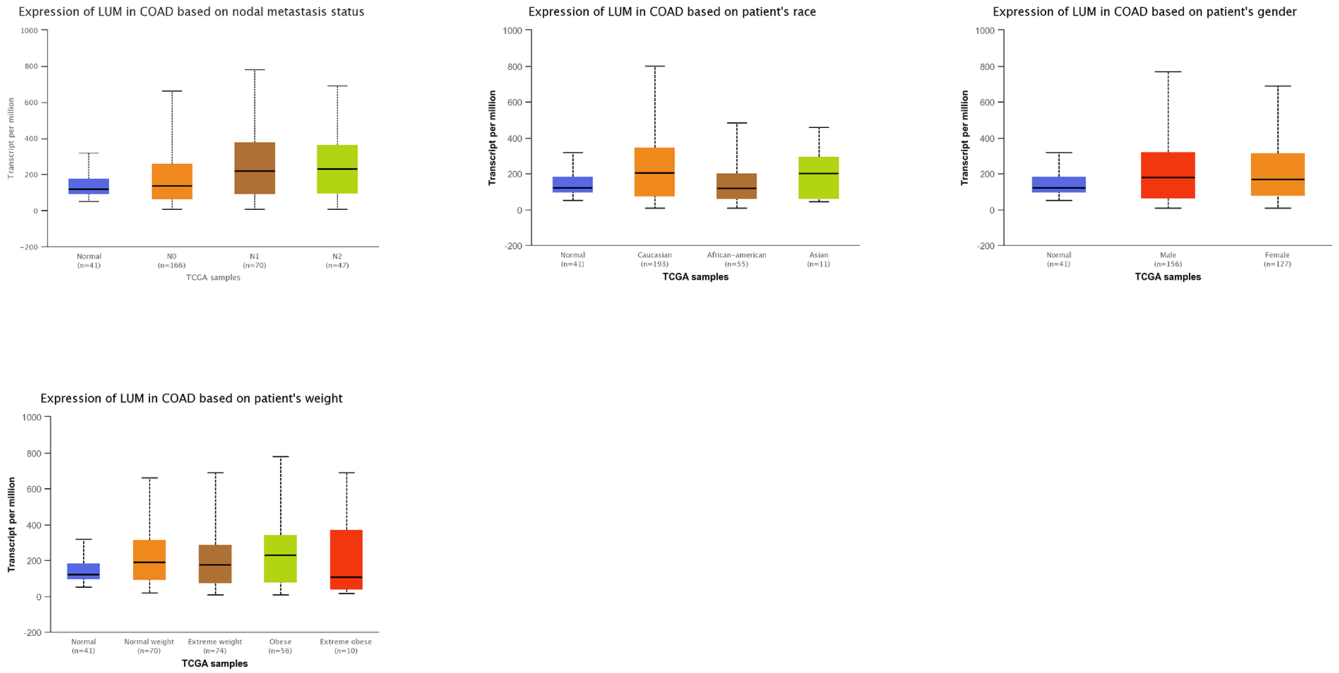

Supplementary Figure 1. The expression of LUM in different clinical information (race, sex, weight and lympyh node metastasis status).

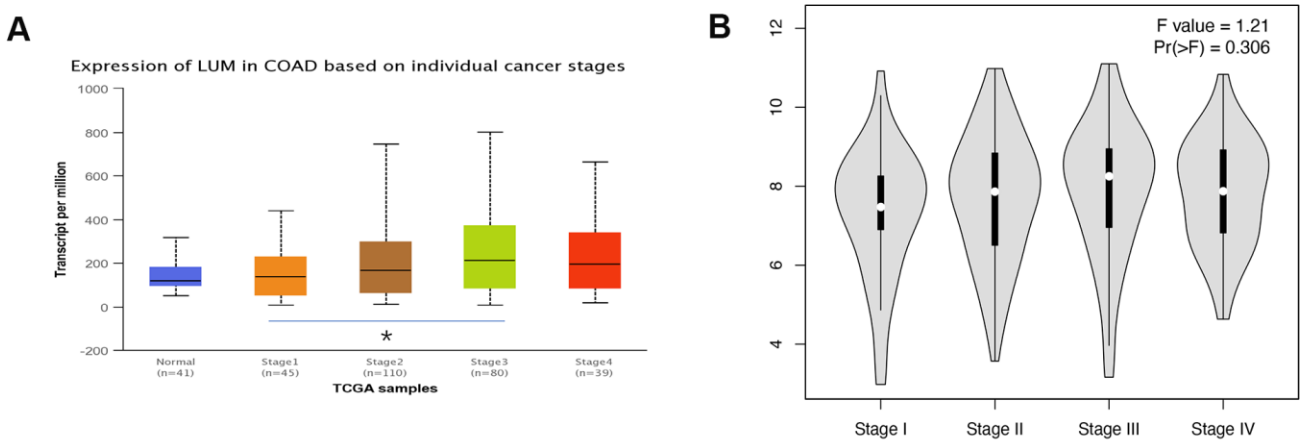

Supplementary Figure 2. LUM expression in COAD based on individual cancer stages. (UALCAN website and GEPIA website). (**A**) LUM expression based on individual cancer stages in UALCAN website. (**B**) LUM expression in COAD based on individual cancer stages in GEPIA.

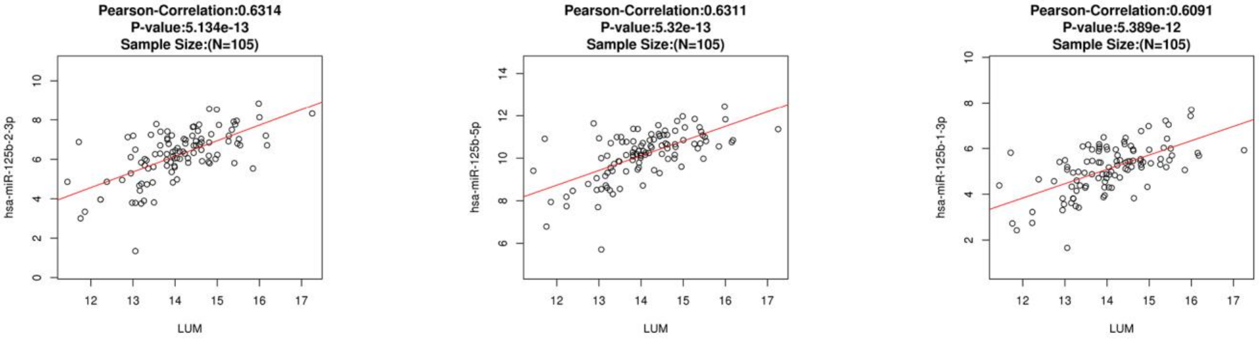

Supplementary Figure 3. The scatter Diagram of the relationship between LUM and miRNa125b Family.

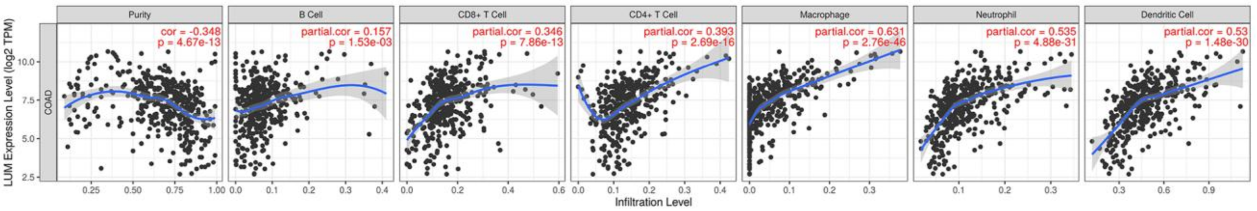

Supplementary Figure 4. The correlation of LUM expression with immune infiltration level in diverse cancer types.

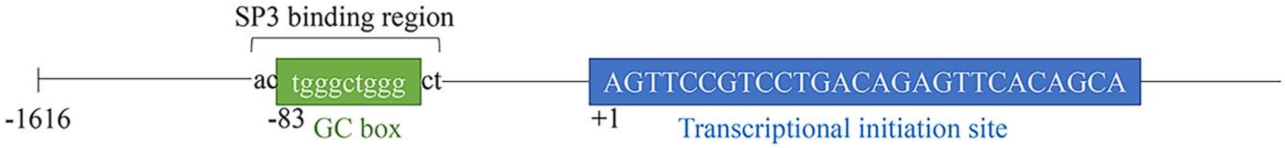

Supplementary Figure 5. Binding region of SP3 and LUM promoter region in JASPAR website.
