# Supplementary material for: Lumican inhibits immune escape and carcinogenic pathways in colorectal adenocarcinoma
Source: Aging (Albany NY). 2021 Jan 20;13(3):4388–408. doi: 10.18632/aging.202401 (PMC7906189; doi:10.18632/aging.202401)
Supplement: Supplementary Table 2 [file aging-13-202401-s003.pdf]

Supplementary Table 2. Partial results of multivariate COX regression.

Case processing summary			
		**N**	**Percent**
Cases available in analysis	Eventa	73	40.10%
	Censored	104	57.10%
	Total	177	97.30%
Cases dropped	Cases with missing values	5	2.70%
	Cases with negative time	0	0.00%
	Censored cases before the earliest event in a statum	0	0.00%
	Total	5	2.70%
Total	182	100.00%	
a.Dependent Variable:time			

Variables not in the Equation			
	**Score**	**df**	**sig.**
X1=LUM	3.893	1	0.048
X2=Age	0.472	1	0.492
X3=Gender	0.177	1	0.674
X4=Stage	51.546	1	0.000
X5=Grade	8.161	1	0.004

Model if Term Removed				
**Term Removed**		**Loss Chi-square**	**df**	**Sig.**
Step1	X4=Stage	54.459	1	0.000
Step2	X2=Age	4.292	1	0.038
	X4=Stage	58.273	1	0.000
Step3	X1=LUM	4.988	1	0.026
	X2=Age	6.185	1	0.013
	X4=Stage	58.475	1	0.000
